# The multidisciplinary management of HER2-positive breast cancer brain metastases: from new biological insights to future therapeutic options

**DOI:** 10.3389/fonc.2024.1447508

**Published:** 2024-12-19

**Authors:** Claudia von Arx, Claudia Calderaio, Alessandra Calabrese, Benedetta Marciano, Claudia Martinelli, Vincenzo Di Lauro, Ivana Cerillo, Daniela Cianniello, Michelino De Laurentiis

**Affiliations:** ^1^ Department of Breast and Thoracic Oncology, Istituto Nazionale Tumori IRCCS Fondazione Pascale, Naples, Italy; ^2^ Clinical and Translational Oncology, Scuola Superiore Meridionale (SSM), Naples, Italy; ^3^ Department of Clinical Medicine and Surgery, University of Naples Federico II, Naples, Italy

**Keywords:** brain metastases, liquid biopsy, antibody-drug-conjugated, TKIs, HER2-positive breast cancer

## Abstract

The advent and success of new drugs for treating HER2-positive metastatic breast cancer has led to a constant improvement in disease and progression-free survival as well as overall survival. Despite these advantages, the overall survival and quality of life of patients with HER2-positive breast cancer brain metastases are significantly worse than the ones of patients with HER2-positive breast cancer metastases outside the brain. For this reason, prevention and treatment of brain metastasis remain a major clinical challenge and the keys to further improving the clinical and survival outcomes of HER2-positive breast cancer patients. This review discusses the etiopathogenesis of brain metastasis, the currently available treatments, and the future perspective on new treatment strategies and diagnostic tools.

## 1 Introduction

One of the most common causes of brain metastases (BM) in oncology is breast cancer (BC). The incidence of BM in BC patients is reported to be around 10% to 16% (1).

The risk of BM is higher in human epidermal growth factor receptor-2 positive (HER2+) or triple-negative (TN) BC. These subtypes of BC account for 30–40% of all metastatic breast cancer (MBC) (2, 3). Retrospective data reveal that almost half of patients with HER2+ or triple-negative BC develop central nervous system (CNS) disease in their lifetime (3–6). In a real-world study of 1012 patients, the median time to first BM appearance was 13.3 months from HER2+ MBC diagnosis (n= 302) (4).

The incidence of BM in these populations has progressively increased over time (7). This is mainly due to three factors: 1. the augmented awareness of BM, 2. more sensitive neuroimaging techniques that enhanced early detection, and 3. the approval of more effective systemic treatment that increases the control of systemic disease and prolongs survival (8).

The management of HER2+ BCBM represents a noteworthy clinical challenge. It requires the involvement of a multidisciplinary team to tailor the optimal treatment sequence, which could include a combination of local interventions, surgical or radiotherapeutic, and systemic treatments (9, 10). To make this more challenging, the recent approval of new drugs has demonstrated strong efficacy in HER2+ BC BM control and has changed the therapeutic algorithm for this group of patients (11).

**Figure 1:**
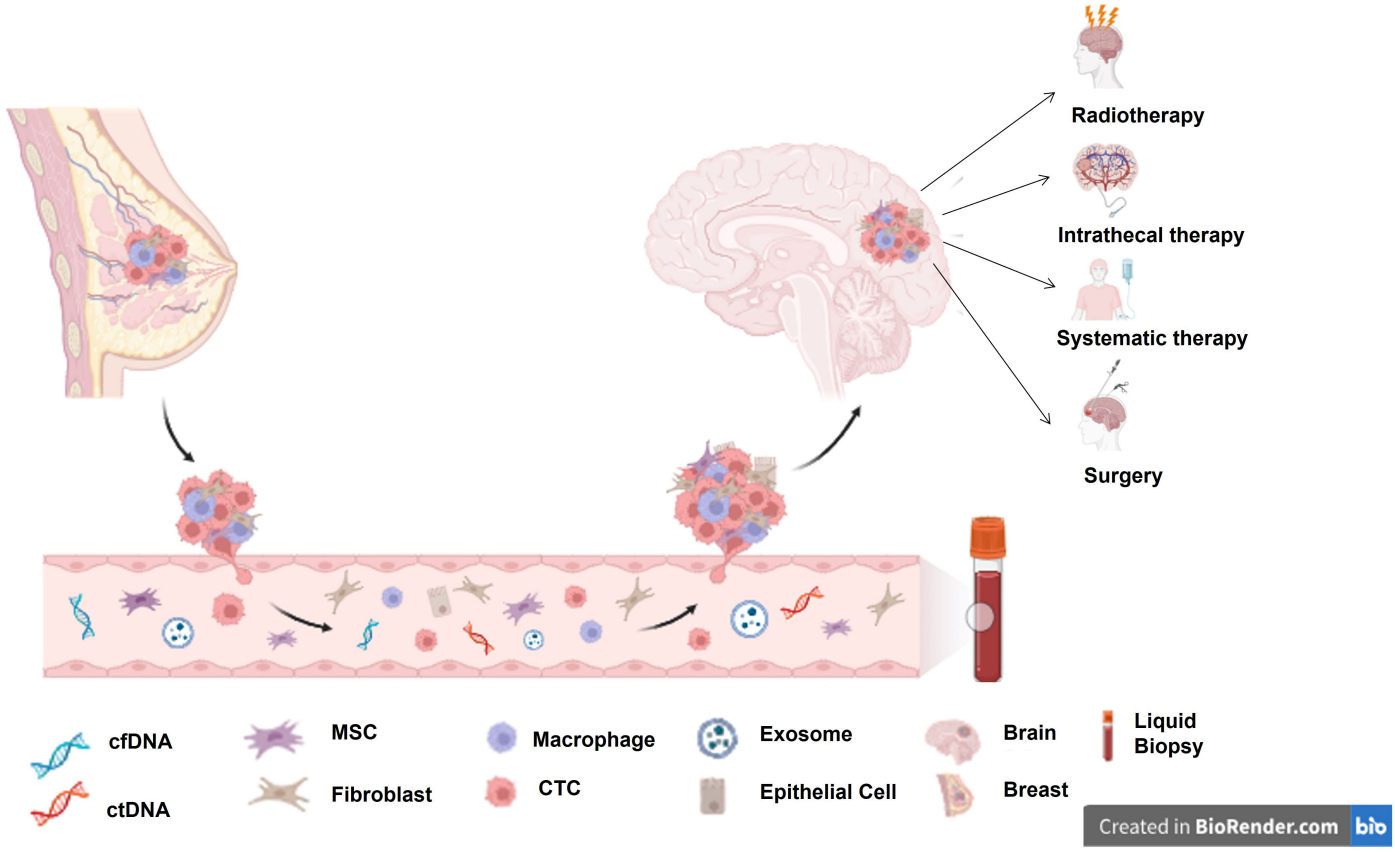
BCBM seeding process and currently available treatment options for BCBM. cfDNA, circulating cell-free DNA; ctDNA, circulating tumour DNA; MSC, Mesenchymal stem cell; CTC, circulating tumour cell.

This review discusses the activity of the therapeutic strategies currently available for patients with HER2+ BCBM. In addition, a specific focus is dedicated to the future directions of research and the discussion of the open questions on BM early diagnosis (**Figure 1**).

## 2 Breast cancer brain metastases pathogenesis

BC metastasis seeding is a complex multistep process. Different factors and pathways determine this process. Metastases result from the selective growth within and release from the primary tumour of subpopulations of cells with enriched survival and invasion characteristics that enable them to survive and complete the metastatic process (12).

BC can metastasise to different organs and tissues (13). However, each BC subtype possesses different gene signatures and relies on various signalling pathways for their growth and dissemination; this implies that each BC subtype has specific metastatic site preferences (14).

Specifically, HER2+ BCs have a higher BM risk than luminal BC (ER+ or PR+).

Organ-specific colonisation is related to the interplay between cancer cells and the local microenvironment and the consequent activation of specific molecular pathways that favour cancer cells in immune evasion and seeding and soiling the organ-specific microenvironment (15, 16).

The CNS’s microenvironment differs radically from that of extracranial lesions because of its distinctive anatomy, cell types, metabolic constraints, and immune environment. A unique cellular barrier tightly controls this microenvironment: the blood-brain barrier (BBB), which allows for proper neuronal function (17).

The BBB consists of a highly selective semipermeable layer of endothelial cells (ECs). The ECs’ tight junctions are responsible for the BBB’s selectivity.

CNS vessels are continuous, non-fenestrated vessels. They own specific properties that tightly regulate CNS homeostasis and protect the brain from external toxic agents and pathogens. This selective BBB needs to be overcome by cancer cells to enter the brain parenchyma (17).

Multiple pieces of evidence suggest that brain metastatic lesions significantly alter the BBB. The metastatic process to the brain passes through an alteration of BBB integrity, permeability, and structural composition13 that transforms the BBB into a highly heterogeneous and variably permeable blood-tumour barrier (BTB) (18–21).

As initial steps of this transformation process, CD31+ endothelial capillaries enlarge and become less dense (18, 19), preexisting blood vessels of BBB undergo vascular remodelling, and local levels of vascular endothelial growth factor increase (20). Additionally, in endothelial and astrocytic basement membranes, levels of basement collagen membrane part IV and laminin α2 decrease (21).

Along with this, pericyte coverage is rearranged, with an increase in the desmin+ pericytes subpopulation and a decrease in the subpopulation CD13+,15+, and 16+, facilitating metastases’ invasion and progression to the brain (22, 23).

These alterations in the functional properties of the BBB are also one factor that determines drugs’ reaching BCBM.

A deeper understanding of the tumour cells and tumour microenvironment interactions in the brain could guide the development of new preventive and therapeutic approaches for BM.

## 3 Therapeutic strategies

The management of patients with BCBM should be multidisciplinary. Fundamentals include identifying the optimal treatment type (local vs systemic vs combined), timing, and sequence. Recently, the treatment of patients with HER2+ has witnessed a drastic change due to the approval of new drugs active at the CNS level.

**Figure 2:**
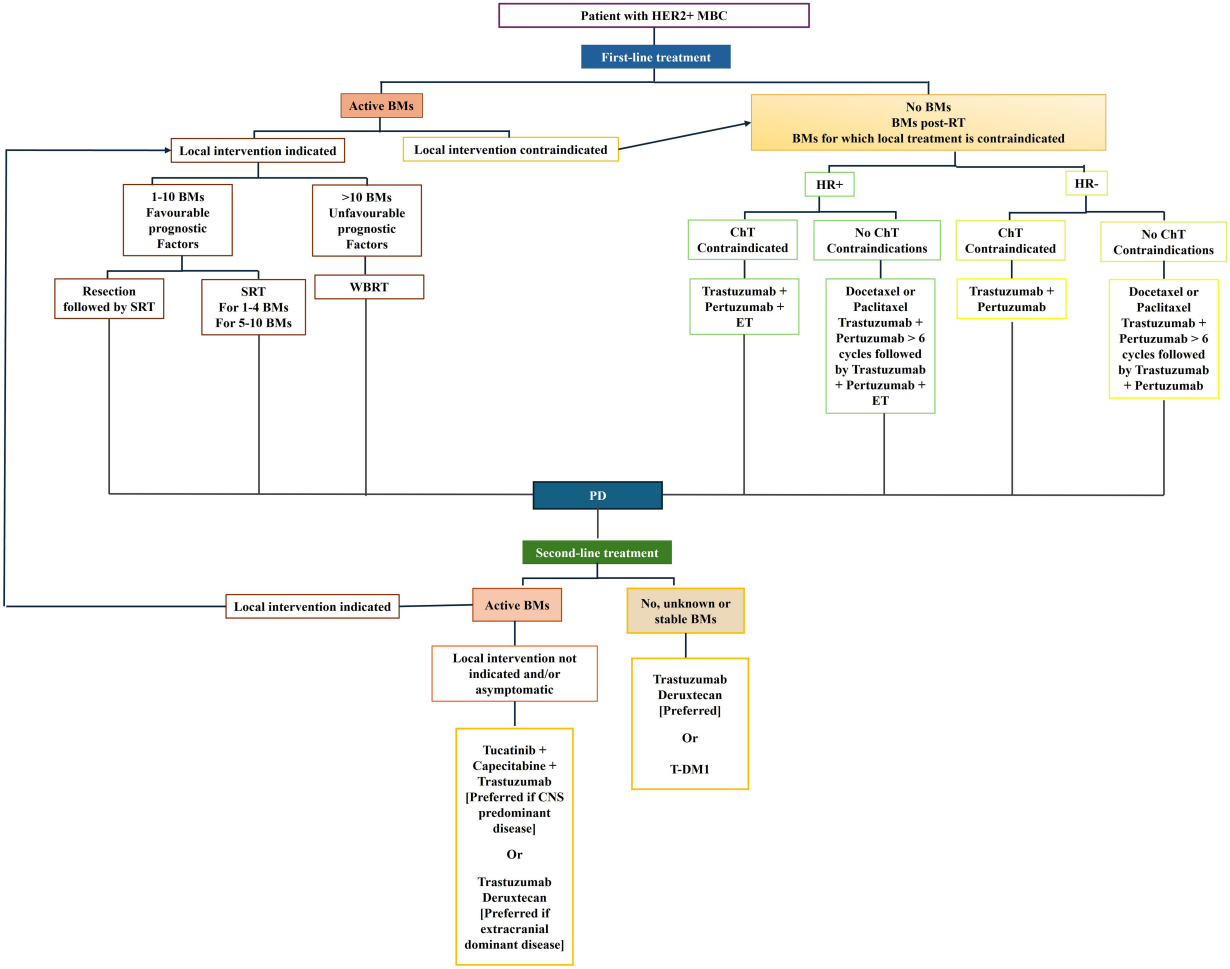
Treatment algorithm of HER2^+^ MBC.

Currently, managing patients with BCBM requires identifying symptomatic vs. non-symptomatic patients because the most updated guidelines indicate that treatment differs between the two groups. The treatment of choice for symptomatic BM is traditionally local. The number of BM, the performance status (PS), and the control of no-CNS metastatic disease guide the choice between neurosurgery and radiotherapy (24). Conversely, systemic treatments are preferred for asymptomatic disease (**Figure 2**).

Furthermore, it is crucial to evaluate whether a patient with active CNS involvement, even if asymptomatic, presents with CNS-dominant versus extracranial-dominant disease. This distinction matters as certain therapies have shown considerable efficacy in reducing extracranial metastases, such as trastuzumab deruxtecan (T-DXd) (25, 26), which is now also being recognized for its activity at CNS level (25, 26). While agents like Tucatinib have demonstrated significant efficacy specifically in treating CNS metastases (27). Consequently, treatment decisions regarding systemic therapy should be tailored to the individual patient, taking into account both the specific BC characteristics and the patient’s overall clinical status.

### 3.1 Local treatment

A good PS, a small number of BM, or large symptomatic lesions (>=3cm) make patients the optimal candidates for surgery as local treatment.

Adjuvant radiation therapy should be evaluated post-BM resection because it offers advantages in terms of survival outcomes, symptom control, and reduction of local recurrence (28–30).

Post-operative stereotactic radiosurgery (SRS) should be the first choice whenever possible. Compared to the postoperative observation, SRS significantly reduced BM local recurrence (31–34).

When surgical resection is not feasible and if the patient is symptomatic, radiotherapy is recommended. Radiotherapy can be SRS or whole-brain radiotherapy (WBRT). The global volume mainly determines the choice of one radiotherapeutic approach over another (34).

SRS is a highly selective radiotherapy in which high-dose radiation is delivered to focused, restricted areas. In the case of intracranial (IC) lesions, the target accuracy of this technique is ∼1 mm.

SRS is indicated in the case of a limited number (1-4) of BM or if the cumulative BM volume is less than 15 ml, even in the case of a higher number (5-10).

Conversely, WBRT is a low-selective way to deliver radiation, which involves radiotherapy delivered to the whole brain.

A low benefit-risk ratio characterises WBRT; therefore, it should be reserved only for patients unsuitable for SRS because of the high number or volume of BM or the specific site of BM (e.g., BM with leptomeningeal involvement) (35). Supportive care should be reserved for patients with poor PS.

### 3.2 Systematic therapy

Systemic therapy is crucial in the long-term control of BCBM. Following the approval of Tucatinib and trastuzumab deruxtecan, the international guidelines have made systemic therapy the first choice in case of asymptomatic HER2+ BCBM (11).

In the context of managing HER2-positive BCBM, it is crucial to distinguish between two essential aspects of systemic therapy. Systemic therapy may function in a dual capacity: first, as a preventative strategy to mitigate the risk of developing BMs, particularly when administered as adjuvant therapy or in the systemic management of MBC outside the CNS. Second, it can be utilized therapeutically to address established BMs. Interestingly, the same pharmacological agents may exhibit both prophylactic and curative properties; however, their efficacy can differ markedly depending on the context of use. The following paragraph will delve into these facets and their clinical repercussions for each approved agent in the treatment armamentarium for HER2-positive MBC.

The trials discussed above are summarised in **Table 1**.

**Table 1:** CNS outcomes in main clinical trials with anti-HER2 agents.

	CLEOPATRA (n=808)	PATRICIA (n=40)	LANDSCAPE (n=45)	EMILIA (n=991)	TH3RESA (n=602)	KAMILLA (n=2002)	NALA (621)	CEREBEL (n=540)
**Trial design**	III R	II	II	III R	III R	IIIb	III R	III R
**Active Treatment**	D+T+P	T hd + P	C + L	T-DM1	T-DM1	T-DM1	C + N	C + T
**Comparator**	D+T	–	–	C + L	–	–	C + L	C + L
**PTS with BM (%)**	0	100	100	9.6%	11.1%	19.9% (6.3% measurable BM)	16.3%	0
**BM inclusion criteria**	Not included	Progressed to previous	Untreated, asymptomatic or symptomatic	Stable, asymptomatic	Stable, asymptomatic, previous RT	Stable, treated with previous RT or untreated, asymptomatic	Stable, asymptomatic	Not included
**Outcome**	Time to BMs as first site of progression: 15.0 vs 11.9 month; HR 0.58 95% CI 0.39–0.85, P = 0.0049. OS in pts with BM as first site of progression: 34.4 vs 26.3 months; HR 0.66 95% CI 0.39–1.11 P = 0.1139	ORR: 11.1% 6mo-CBR: 51%	Objective CNS response:65.9% Objective CNS response (RECIST): 57% Improvement of symptoms: 58% TTP: 5.5 months 6-months OS: 90.9% Median OS: 17.0 months	Median PFS: 5.9 vs 5.7 months HR: 1.00 95% CI: 0.54–1.84 P=1.00 Median OS 26.8 vs 12.9 months HR 0.3	Median PFS: 5.8 vs 2.9 months HR: 0.47 95% CI: 0.24–0.89 P = NA	Median PFS: 5.5 months Median OS: 18.9 months ORR (all sites): 21.4% CBR (all sites): 42.9% ORR (on CNS disease): 42.9%	Cumulative incidence of any intervention for CNS disease: 22.8% vs 29.2% HR: 0.78 95% CI: 0.60–1.01 P = 0.043	CNS metastases at first site of relapse 5% vs 3% Incidence of CNS progression= 6% vs 7% Median time to CNS progression= 4.4 vs 5.7 months

BMs, Brain metastases; C, Capecitabine; CNS, Central Nervous System; CBR, Clinical Benefit Rate; D, Docetaxel; hd, high-dose; HR, Hazard Ratio; L, Lapatinib; N, Neratinib; NA, not Applicable; ORR, Overall Response Rate; OS, Overall Survival; P, Pertuzumab; Pbo, Placebo, PFS, Progression Free Survival; pts, patients; R, randomized; RT, radiotherapy; T, Trastuzumab; T-DM1, Trastuzumab emtansine; T-DXd, Trastuzumab deruxtecan; TPC, Treatment of Physician Choice; TTP, Time to Progression; Tuc, Tucatinib.The bold values were inserted for graphical reasons.

#### 3.2.1 Monoclonal antibodies

Trastuzumab and pertuzumab are FDA-approved HER2-targeted monoclonal antibodies (mAbs) used to manage metastatic HER2-positive breast cancer. While a robust body of evidence supports their efficacy in preventing the development of brain metastases (BCBM), studies assessing their intracranial efficacy remain limited.

##### 3.2.1.1 Trastuzumab

Trastuzumab is a humanised recombinant monoclonal antibody that targets the extracellular domain (subdomain IV) of the HER2 protein and blocks the homodimerisation of the HER2 receptor.

Adding trastuzumab to standard chemotherapy has significantly improved the survival outcomes of both HER2+ early BC (EBC) and MBC patients and is the standard treatment in both settings (36–41).

Although trastuzumab has shown positive effects on the survivals of HER2+ EBC, CNS recurrence still represents a major health issue (42).

The role of trastuzumab in preventing and improving the outcomes of patients with BCBM has been evaluated only in *post hoc* and retrospective trials.

Two retrospective trials derived the preventive effects of trastuzumab on BM occurrence. Both demonstrated that patients with HER2+ BC who underwent trastuzumab treatment experienced a significantly longer median time to BCBM than patients who did not, with a gain of 11 months (HR 2.13, 95% CI 1.51–3.00, p = 0.28) (43, 44).

Several studies also demonstrated that trastuzumab prolongs survival outcomes in patients with BCBM. From these studies, the median OS (mOS) in patients with HER2+ BCBM who received trastuzumab-based treatments was higher compared with mOS of patients who did not receive trastuzumab, ranging respectively from 9.0 to 26 months vs 2.0 months to about 9 months (4, 43, 45–50).

Although the benefits derived from trastuzumab treatment, patients with BCBM still have the worst clinical outcomes compared to patients with MBC to organs outside the CNS. Therefore, other strategies have been tested further to improve the clinical outcomes of patients with BCBM. One of these strategies is to increase intracerebral trastuzumab concentration.

Trastuzumab’s dose-dependent activity has been demonstrated in IC tumour models (51). Therefore, a higher dose of trastuzumab (6 mg/kg weekly) combined with pertuzumab was tested in the phase II PATRICIA trial in HER2+ MBC patients with progressive BM after radiotherapy (52). The CNS lesions disease control rate was 68%; however, the overall response rate (ORR) was limited.

Other techniques to increase IC trastuzumab concentration include an intrathecal or a super-selective intra-arterial cerebral infusion of trastuzumab.

The safety and activity of intrathecal trastuzumab for leptomeningeal metastases have been reported in phase I/II trials, numerous case reports, and retrospective cohort studies. In all these reports, intrathecal trastuzumab showed a good safety profile and a discrete benefit (53, 54).

A trial investigating intra-arterial cerebral trastuzumab infusion is ongoing in patients with HER2+ BCBM (NCT02571530).

##### 3.2.1.2 Pertuzumab

Pertuzumab is a recombinant human monoclonal antibody that prevents HER2 from merging with other HER family receptors by binding HER2 extracellular subdomain II (55).

Therefore, pertuzumab acts synergistically with trastuzumab.

The CLEOPATRA phase III trial results made pertuzumab to be approved as the first-line treatment of HER2+ MBC. In this study, the addition of pertuzumab to the standard first-line treatment, trastuzumab and taxanes, showed a significant improvement in progression-free survival (PFS) (HR 0.68, P < 0.001) and OS (HR: 0.68, P < 0.001) (56).

In the CLEOPATRA study, the presence of BM was an exclusion criterion. However, an exploratory analysis showed that pertuzumab prolonged by 3.1 months the median time to CNS metastases HR 0.58, 95% CI 0.39-0.85, p= 0.0049) (27).

These results suggest the role of pertuzumab in BM prevention.

Data concerning the intracranial efficacy of Pertuzumab predominantly stem from a retrospective trial assessing the intracranial response in HER2-positive patients with brain metastases (BCBM) treated with concomitant trastuzumab and pertuzumab. Notably, this cohort’s intracranial overall response rate (IC-ORR) reached 92.9%. However, it’s important to note that 88.5% of these patients also received local treatments either concurrently or prior to initiation of the pertuzumab/trastuzumab regimen. This suggests that the reported efficacy may reflect a synergistic effect rather than isolating the intrinsic intracranial activity of Pertuzumab alone (57).

#### 3.2.2 Antibody-drug conjugates

Antibody-drug conjugates (ADCs) are sophisticated therapeutic agents composed of mAbs linked covalently to cytotoxic agents through a chemical linker. This design harnesses the specificity of mAbs for targeted delivery to neoplastic cells while simultaneously utilising the drug’s potent cytotoxicity. This dual mechanism facilitates the precise and effective eradication of cancer cells, positioning ADCs at the forefront of anticancer drug development and research.

Ado-Trastuzumab Emtansine (T-DM1) and Trastuzumab deruxtecan (T-DXd) are currently the two FDA-approved anti-HER2 monoclonal antibodies for managing HER2-positive metastatic breast cancer (MBC). Among these, T-DXd has the most extensive clinical evidence demonstrating its efficacy not only in the prevention but also in the treatment of BMs.

##### 3.2.2.1 Ado-trastuzumab emtansine

Trastuzumab emtansine (T-DM1) was the first antibody-drug conjugate (ADC) approved for HER2+ BC. T-DM1 consists of trastuzumab covalently linked via a stable thioether linker with the antimicrotubule agent DM1. Therefore, the DM1 is precisely delivered to the HER2-expressing cells (58).

Based on the randomised phase III EMILIA trial results, T-DM1 has been approved as second-line treatment after the failure of trastuzumab and pertuzumab in HER2+MBC (59). This study compared T-DM1 and the combination of lapatinib-capecitabine in HER2+ MBC metastatic patients who progressed after taxane and trastuzumab treatment.

T-DM1 significantly prolonged median PFS (9.6 months versus 6.4 months; HR 0.65, P<0.001) and OS (HR = 0,68, P < 0,001) over lapatinib and capecitabine.

In a secondary analysis of the EMILIA trial, the efficacy of T-DM1 vs. lapatinib-capecitabine was evaluated in patients with BM. Retrospectively, 95 patients enrolled in the EMILIA trial had asymptomatic CNS metastases. T-DM1 achieved a significantly longer OS in this subpopulation than lapatinib and capecitabine (26.8 vs. 12.9 months, HR = 0.38, P = 0.008) (60).

A subgroup analysis of the KAMILLA trial further demonstrated the efficacy and safety of T-DM1 in patients with HER2+ BCBM. T-DM1 achieved a BM response rate of 21% in the 398 patients with BM enrolled in the trial, making register 6 months and 19 months of mPFS and mOS (61).

Several retrospective studies confirmed the efficacy of T-DM1 on BM treatment, with documented BM response rates up to 44% (62).

##### 3.2.2.2 Trastuzumab deruxtecan (DS8201)

Trastuzumab deruxtecan (T-DXd) is a second-generation ADC composed of trastuzumab linked by a cleavable linker with DXd, a cytotoxic topoisomerase I inhibitor. The higher antibody: cytotoxic ratio (1:8) of T-DXd compared to T-DM1 and the highly membrane-permeable payload make T-DXd more efficient in the presence of lower HER2 expression.

In the DESTINY-Breast01 trial, T-DXd demonstrated a durable antitumor activity in a pretreated patient population with HER2+-MBC. Trastuzumab deruxtecan achieved an RR of 60.9% (95% CI, 53.4-68.0), with 6.0% of patients having a complete response (CR) and 54.9% a partial response (PR). The median time to progression (TTP) was 16.4 months (95% CI, 12.7 to not reached). A good TTP of 18.1 months (95% CI, 6.7 to 18.1) was also achieved in the 24 enrolled patients with treated and asymptomatic BM at baseline (25).

DESTINY-Breast03 (DB03) trial results made T-DXd to be approved as second-line treatment for MBC patients after one anti-HER2-based regimen failure. In this trial, T-DXd obtained a significantly longer mPFS (28.8 months) compared to T-DM1 (6.8 months) (HR= 0·33; 95% CI 0·26-0·43; p<0·0001) in HER2+ MBC progressed on a first-line therapy with trastuzumab and taxane. The same superiority was also registered in terms of mOS (HR=0·64; 95% CI 0·47-0·87; p=0·0037) (26). In the DB03 trial, 82 patients with stable BMBC were included. Data from this subgroup was presented at the San Antonio Breast Cancer (SABCS) meeting. In patients with BM at baseline (n=82), median PFS was 15 months with T-DXd vs 3 months with T-DM1: a 75% improvement favouring T-DXd. Among these patients, the confirmed ORR for T-DXd was 67.4% vs 20.5% for T-DM1 (25, 26).

At the European Society for Medical Oncology Congress 2023, a pooled analysis of DESTINY-Breast01, -02 and -03 was presented to assess the efficacy of T-DXd in patients with BCBM. One-hundred-forty-eight patients who received T-DXd had BMs at baseline; 104 (70.3%) had treated BMs, and 44 (29.7%) had untreated BMs; 16.3% of patients with treated/stable BMs had a CR. 15.9% of patients with untreated/active BMs had a CR. IC- ORR was 45.2% in the treated/stable BMs group and 45.5% in the untreated/active BMs group.

Other trials evaluated the role of T-DXd in treating active BMs. The TUXEDO-1 trial was a single-arm prospective trial in patients with pre-treated trastuzumab and pertuzumab and newly diagnosed untreated BM or active BM progressing after previous local therapy, with no indication of immediate local treatment. T-DXd achieved a RR of 73.3% (11/15). The median PFS was 14 months (95% CI 11.0 months-NA). The median PFS was maintained in all patient subgroups (previous BM treatment, T-DM1 therapy, hormone receptors positive vs negative and ECOG). Only three dead were registered at the 12-month data cut-off. Therefore, the median OS was not reached (63).

A Japanese real-world study (UMIN000044995) consolidated the role of T-DXd in patients with HER2+ BC with BM or leptomeningeal carcinomatosis. The trial demonstrated an IC-ORR of 62.7% (95% CI, 48.1%-75.9%), an intracranial stability rate of 31.4%, and intracranial progressive disease rates of 5.9% among the 59 patients with IC disease. The overall 6-month IC clinical benefit rate (CBR) in this subpopulation was 70.6% (95% CI, 56.2%-82.5%) (64).

The ongoing DEBBRAH trial (NCT04420598) evaluates T-DXd efficacy in patients with HER2+ and HER2-low BC CNS metastases.

Patients with stable, untreated, or progressing BMs from HER2+ or HER2-low pre-treated MBC could be enrolled in this five-cohort phase II study. Preliminary results showed that T-DXd achieved a 16-week PFS rate of 87.5%; the IC-ORR of HER2+ BC patients with asymptomatic untreated BM was 50.0% and 44.4% for patients with progressive BM. The overall IC-RR was 46.2% (asymptomatic untreated + progressing BMs) (65).

Lastly, at ESMO 2024 were presented the data on patients with HER2-positive BCBM treated with T-DXd in the context of the ongoing DESTINY B12 (NCT04739761) phase 3b/4 multicenter trial. These patients could have received up to 2 lines of therapy in the metastatic setting, and have stable or active BMs. In patients with BMs, the 12-month PFS rate was 61.6% (95% CI, 54.9%-67.6%) and the 12-month central nervous system PFS was 58.9% (95% CI, 51.9%-65.3%). The rates were similar in patients with stable (57.8%; 95% CI, 48.2%-66.1%) and active (60.1%; 95% CI, 49.2%-69.4%) BMs. These results demonstrated significant intracranial activity of T-DXd (66).

#### 3.2.3 Tyrosine kinase inhibitors

TKIs, as small molecules, have shown significant success in treating HER2+ BCBM due to their ability to potentially penetrate the blood-brain barrier (BTB) and simultaneously inhibit multiple receptors within the ErbB2 family. Currently, three TKIs are approved for HER2+ MBC: lapatinib, neratinib, and tucatinib. Of them, tucatinib demonstrated better outcomes.

##### 3.2.3.1 Lapatinib

Lapatinib is an oral reversible inhibitor of epidermal growth factor receptor (EGFR) and HER2 TKs (67).

In a phase II trial, Lapatinib plus capecitabine achieved an IC-ORR of 30% in patients with WBRT-pre-treated BM (68, 69).

In the LANDSCAPE trial (single-arm phase II), the efficacy of lapatinib plus capecitabine was further confirmed in the first-line setting. The combination achieved a 65.9% CNS ORR, 5.5 months of median time to CNS progression and 8.5 months to WBRT in 45 patients with untreated low-volume BM (70).

Despite these results, a subgroup analysis of the EMILIA trial did not demonstrate the superiority of lapatinib and capecitabine over T-DM1 in treating established BM. 52.

Furthermore, in terms of BM prevention, first-line or second-line treatment with lapatinib and capecitabine was not superior to trastuzumab plus capecitabine (3% vs 5%, P = 0.36) as for the results of the CEREBEL trial (71).

##### 3.2.3.2 Neratinib

Neratinib is an oral, irreversible pan HER TKI (72).

Neratinib plus capecitabine demonstrated an ORR of 49% (18 patients) and a 6-month DCR of 19% (7 patients) in lapatinib naïve (n = 37) patients with HER2+ BCBM enrolled in the phase II TBCRC 022 trial. In this population, the median PFS was 5.5 months, and the median OS was 13.3 months (73).

In the NALA study, neratinib plus capecitabine reduced the’ overall cumulative incidence’ of intervention for BM (mostly RT) by 7% (from 29.2% to 22.8%; p = 0.043) compared to lapatinib plus capecitabine in 130 patients with HER2+ BMBC in the second/third line of treatment (74).

In the open-label NEfERT-T trial, neratinib plus paclitaxel was compared with trastuzumab plus [paclitaxel as first-line treatment for recurrent or metastatic HER2+; in these trials, patients with asymptomatic CNS metastases were allowed to enroll (75). Median PFS was the same in both arms (12.9 months vs 12.9 months). However, neratinib-paclitaxel was superior in reducing the incidence (HR: 0.48; 95% CI, 0.29-0.79; p = 0.002) and delaying time to CNS metastases (HR, 0.45; 95% CI, 0.26-0.78; p = 0.004).

A phase II trial for HER2+ BCBM patients (NCT01494662) is evaluating the treatment with neratinib plus T-DM1 patients.

##### 3.2.3.3 Tucatinib

Tucatinib is an oral TKI that reversibly inhibits HER2. In 2020, the FDA approved the combination of tucatinib, capecitabine, and trastuzumab as ≥ a second-line treatment in patients with inoperable or metastatic HER2+ BC.

Tucatinib is the only anti-HER2 treatment that has demonstrated a benefit in HER2+ BCBM outcomes in a study with specific BM endpoints (28).

In light of the results of a phase I trial, which demonstrated a notable efficacy of tucatinib + capecitabine + trastuzumab in BCBM control (76), the HER2CLIMB trial has been designed to assess the effectiveness of tucatinib added to trastuzumab and capecitabine in patients with HER2+MBC. Of the 291 patients with HER2+ BMBC enrolled in the trial, 174 had active BM, and 117 had stable BM after a previous treatment.

The addition of Tucatinib achieved a higher CNS ORR than the control arm (47% vs 20%, p = 0.03), a 3.8months longer median duration of response, and a 5.7 months gain in median CNS PFS (p < .00001). The risk of progression or death in patients with BM was significantly reduced by 68% in the tucatinib arm (HR 0.32, P < 0.00001), with a significant prolongation of mOS in the subgroup of patients with BM (18.1 versus 12.0 months). The registered advantages were similar in both patients with active and stable BM populations (28).

The HER2CLIMB02 trial compared T-DM1 alone to T-DM1 plus Tucatinib. Preliminary results were presented at the SABCS meeting in December 2023 (77).

Two hundred four patients with BM were enrolled in this trial, 107 with active BM and 97 with treated and stable BM. Adding tucatinib to T-DM1 improved the median PFS in this population (7.8 months vs 5.7 months; HR 0.64). mOS was not yet reached (77).

## 4 Future perspective

The management of brain metastases is poised to evolve by integrating new therapeutic algorithms, driven by the recent approval of and the advent of new CNS-active pharmacotherapies, and the development of enhanced diagnostic modalities, which will facilitate earlier detection of brain metastases and, ideally, allow for the proactive identification of patients at risk for developing these metastases.

Many trials are ongoing to evaluate new drugs in association with already approved anti-HER2 treatment and to identify new strategies for prevention and early diagnosis (**Table 2**).

**Table 2:** Ongoing trials in HER2-positive BCBM.

NCT Identifier	Phase	Treatment	Biomarkers
NCT04030507	II	**Preventive:** Screening MRI of the brain in MBCs	All subtypes
NCT05115474	II	Screening brain MRIs in stage IV breast cancer	All subtypes
NCT03994796	II	Genetic testing in guiding treatment for patients with BMs	All subtypes
NCT03617341	II	Brain Monitoring for High Risk of Brain Metastases in Metastatic Breast Cancer	All subtypes
NCT03933982	II	Pyrotinib + vinorelbine	HER2+
NCT04639271	II	Pyrotinib + trastuzumab + Nab paclitaxel	HER2+
NCT01494662	II	Preoperative neratinib with or without capecitabine or T-DM1	HER2+
NCT04760431	II	Taxanes + Trastuzumab + Pertuzumab *vs* Taxanes + Trastuzumab + TKI (neratinib or tucatinib; HER2BRAIN)	HER2+
NCT05323955	II	HP or T-DM1 + tucatinib	HER2+
NCT04512261	II	Tucatinib + trastuzumab + pembrolizumab (TOPAZ)	HER2+
NCT04739761	III	T-DXd	HER2+
NCT04760431	II	Taxanes + Trastuzumab + Pertuzumab *vs* Taxanes + Trastuzumab + pyrotinib	HER2+
NCT05018702	II	ARX788	HER2+
NCT04539938	II	T-DXd, tucatinib	HER2+
NCT03190967	I/II	Metronomic temozolomide and T-DM1	HER2+
NCT03765983	II	Paxalisib (GDC-0084) + trastuzumab	HER2+
NCT04348747	II	Anti-HER2/HER3 dendritic cell vaccine ID, celecoxib, interferon alfa-2b followed by pembrolizumab	HER2+
NCT04158947	II	Afatinib, T-DM1	HER2+
NCT04509596	I	DZD1516 with capecitabine or T-DM1	HER2+
NCT05593094	I	ZN-A-1041 or ZN-A-1041 combination	HER2+
NCT03714243	NA	HIFU (ExAblate BBBD)	HER2+

MBCs, Metastatic Breast Cancers; MRI, Magnetic Resonance Imaging; BMs, Brain metastases; T-DM1, Trastuzumab emtansine; T-DXd, Trastuzumab deruxtecan.

The bold values were inserted for graphical reasons.

### 4.1 New drugs

#### 4.1.1 TKIs under development

Afatinib, an HER2 and EGFR inhibitor, is under investigation in phase I of the dose-finding trial (NCT02423525) in HER2+ BM. Furthermore, it is under evaluation in a phase II trial (NCT02768337) that will assess whether or not the Afatinib penetrance into BM combined with low-dose targeted radiation post-BM surgery. The HER2BAT phase I/II trial tests T-DM1 mono-therapy vs T-DM1 combined with afatinib (NCT04158947).

The combination of Pyrotinib, a HER1-2-4 inhibitor, and vinorelbine is under evaluation in a phase II trial for HER2+ BM patients (NCT03933982).

#### 4.1.2 Targeted therapy

Numerous studies are ongoing to evaluate targeted therapies beyond TKIs further. Among the drugs studied are CDK4/6, PI3K, and ATM inhibitors.

CDK4/6 inhibitors have generated much interest for their capacity to cross the BBB. The primary endpoint of the phase II study NCT02308020 was to assess IC-ORR in patients receiving abemaciclib with brain or leptomeningeal metastases (LM) secondary to HR+mBC. However, this study did not reach its goal; therefore, further studies are warranted.

Palbociclib is being investigated as monotherapy or in combination with trastuzumab, lapatinib, and fulvestrant in two single-arm phase II trials in patients with HER2+ BCBM (NCT02774681, NCT04334330).

A PI3K inhibitor, GDC-0084, is currently under investigation in a phase II trial enrolling patients with HER2+ BCBM (NCT03765983) in combination with trastuzumab.

#### 4.1.3 Immunotherapy

BC has traditionally been regarded as immunologically silent. Several pieces of evidence have recently supported that a subsection of BCs can stimulate the immune system, and some breast tumours have a considerable lymphocytic infiltration (78). This lymphocytic infiltration is found within the tumour and is characterised by a high density of tumour-infiltrating immune (TILs) cells that occupy ≥ 50% of the tumour bed. The proportion of TILs is known to be directly correlated with the prognosis (79).

Several trials are investigating the efficacy and safety of adding immune checkpoint inhibitors to anti-HER2 treatment.

Pembrolizumab plus trastuzumab are currently being investigated in a phase Ib/II trial (PANACEA-trial). The trial is enrolling patients with trastuzumab-resistant PD-L1+ HER2+ MBC (80).

A similar trial, the PembroMab (phase Ib/II) trial (NCT02318901), evaluates pembrolizumab plus trastuzumab or T-DM1 safety and efficacy. Patients with HER2+ MBC are enrolled in this trial irrespective of PDL1 status.

The safety and effectiveness of the combination atezolizumab, paclitaxel, trastuzumab, and pertuzumab are currently under investigation in a single-arm phase II trial in patients with HER2+ locally advanced, unresectable or metastatic BC (NCT03125928).

Several other combinational strategies, including PD-1/PD-L1 inhibitor treatment, are under evaluation. Specifically, regarding patients with BM, the combination of immuno-therapy with SRS is under investigation in three clinical trials: a phase I trial with nivolumab (NCT03807765), a phase II trial with atezolizumab (NCT03483012) and a phase I/II trial with pembrolizumab (NCT03449238). These trials evaluate whether combining immunotherapy and radiotherapy could increase the abscopal effect.

### 4.2 New diagnostic tools

An essential clinical goal is the prevention or early detection of BM in addition to treating patients with BM. However, international guidelines do not recommend brain screening for BC patients because of contrasting data on its potential benefit. Four studies (NCT03881605, NCT04030507, NCT0361734 and NCT00398437) are investigating the potential benefits of systematic radiological screening for early detection.

In addition, the approval of new drugs significantly active at the CNS level that can delay the WBRT has made the early identification and diagnosis of BM crucial. Thanks to the advances in neuroimaging techniques, the identification of BM is becoming more sensitive, even in the case of low BM dimensions (81). However, it does not add molecular and biomarker-specific information that can be only obtained by the histopathological analysis of tumour tissue. The availability of BM tissue requires brain surgery, which is complex and risky. Since genomic characterisation of tumour tissue is essential to cancer diagnosis and treatment, liquid biopsy (LB) is extremely interesting, especially in gaining information on metastasis in complex access sites (82–84).

LB is a non-invasive and simple procedure that allows dynamic observation of tumour characteristics and drivers through the detection of circulating tumour cells (CTCs), circulating tumour DNA (ctDNA), circulating cell-free DNA (cfDNA), and free circulating nucleic acids (NAs) (mRNA and non-coding RNA) in the blood or cerebrospinal fluid (CSF) (82, 83).

cfDNA includes various forms of DNA freely circulating in body fluids. It can derive from cancer cells but is not limited to that. Although cfDNA levels increase in tumours, other conditions or the presence of mutations-derived clonal haematopoiesis of indeterminate potential could be confounding (85).

Conversely, ctDNA, a subset of cfDNA, derives specifically from the tumour, particularly from CTCs undergoing apoptosis and CNS tumour NAs entering biofluids by crossing the BBB (86).

Because of cancer derivation, the concentration of ctDNA in biofluids is generally low. In plasma samples, ctDNA is estimated to be <1% of all cfDNA, and this is even lower in EBC, where disease burden is typically lower (87). This low concentration represents a limit in ctDNA detection and makes methods for that detection highly specific but poorly sensitive. Another limitation is the unequal release of ctDNA/cfDNA from the primary tumour and metastases, raising uncertainty about whether the alterations detected in ctDNA accurately reflect tumour heterogeneity. Despite these limitations, ctDNA detection methods are constantly improving, increasing the detection sensitivity (88).

Currently, ctDNA is used to identify genomic alterations and epigenetic signatures that may anticipate prognosis, monitor response to treatment and identify therapeutic targets in CNS tumours. Still, it has not yet been used to obtain the same information in BMs.

CTCs are a rare subgroup of cancer cells that enter biofluids from solid tumours (89, 90).

CTCs circulation time is almost 1–2.5 hours before degradation by the immune system; however, a small fraction of CTCs can survive and lead to cancer progression by seeding and growing in distant metastatic sites (91). Being precursors of cancer progression, CTCs detection can allow early detection and the identification of progressive disease. This is particularly useful in malignancies, such as CNS tumours, for which re-biopsy is risky (92).

A study of CTCs in MBC evaluated the correlation between the number of CTCs and clinical outcomes, demonstrating that a high number of CTCs (>5 per 7.5 ml of whole blood) before treatment is an independent predictor of shorter PFS and OS (93). In addition, monitoring CTCs levels during treatment allows early evidence of resistance to therapy (94).

Despite the promising prognostic role of CTCs, their clinical application is limited by the challenges of the isolation process. To standardise this expensive and challenging sequencing process, the CellSearch system was granted FDA approval as a clinical method to be used in MBC patients. As for ctDNA, another challenging issue is whether CTCs are indicative of full tumour heterogeneity since there is uncertainty about the equal and uniform shedding of primary tumours and metastases (95).

Besides evaluating CTCs levels, which do not have a fundamental role in detecting BCBM, CTCs can provide DNA, RNA and protein that can conversely give information on metastasis localised in sites challenging to reach with biopsy. In addition, CTCs can be cultured and studied to predict the pathway of metastasis dissemination and, in the case of specific gene signatures, enable early detection of metastatic disease with a specific tropism for the brain (96). This is because BM could derive from primary tumours by disseminating CTCs with particular characteristics into the blood.

Zhang et al. identified a specific BM molecular signature in CTCs isolated from 38 BC patients, comprising HER2+/EGFR+/HPSE+/Notch1+ pathways markers. Suggesting the use of these markers for the specific targeting of BM-initiating CTCs (97). They also demonstrated that CTCs associated with BMs are characterised by increased activity of Notch signalling pathways, pro-inflammatory chemokines, immunomodulatory networks and mitogenic growth factors (98).

Molecular profiling of CTC lines derived from BC patients showed an overexpression of MYC and a copy-number gain of SEMA4D, a BBB transmigration mediator in these CTCs. Therefore, these are identified as novel markers for BM (96).

The recent research on CTCs in BM and the identification of specific biomarkers could push forward the role of CTCs as anticipators of BM and, therefore, the role of their targeting in reducing the risk of BM development (96).

Another diagnostic tool that could be essential in characterising BM without tissue biopsy is the isolation of EVs. EVs are membrane-bound vehicles released by almost all types of cells. They regulate the mediation of intercellular communication, remodelling of membranes, recycling, and elimination of cellular components (83, 84, 99, 100). A diverse array of particles sourced from parental cells can be enclosed inside the EVs. These include proteins, mRNA, circRNA, miRNA, lncRNA, lipids and DNA; among these, the macromolecules of higher interest when EVs are used as liquid biopsy biomarkers are RNA, proteins, and microRNA (miRNA) are (101).

EVs have recently gained popularity as liquid biopsy biomarkers because they offer numerous advantages compared to other liquid biopsy molecules. First, EVs exist in nearly all body fluids. Secondly, EVs have good biological stability, enabling storage at various temperatures (101).

Moreover, EVs could mirror genuine biological mechanisms of metastatic dissemination since EV contents originate from viable parent cancer cells, unlike circulating tumour DNA (ctDNA) (102). These EVs can express surface proteins unique to their originating cells, facilitating the identification of organ- or tumour-specific exosomes and the anticipation of organ-specific metastases (103). Additionally, exosomal DNA exhibits superior sensitivity and specificity compared to ctDNA in detecting mutational frequencies, potentially serving as a prognostic biomarker (104–106).

LB are crucial instruments for continuous sampling throughout a patient’s treatment, offering real-time insights into the tumour-evolving mutation profile. Furthermore, they can act as predictive indicators for precision medicine. NGS methods can be used to construct cancer mutation profiles and develop patient-specific panels for tailoring treatment.

## 5 Conclusions

BMs represent a devasting event for patients with HER2+ MBC and remain an unsolved requirement, given that all these patients will inevitably necessitate local treatments, which could lead to potentially distressing and debilitating consequences.

HER2+ MBC patient treatment is rapidly evolving, with the approval of new drugs that can delay invalidating treatment, such as WBRT. However, further studies are required to understand the resistance mechanisms to these therapies better and to allow treatment-sequencing optimisation.

One of the ongoing debates is whether clinicians should persist with the current treatment approach in the event of oligo progression at the cerebral level following locoregional therapy. Alternatively, the introduction of highly effective agents that not only treat but also prevent further intracranial progression raises the question of whether a therapeutic switch to these agents is warranted.

On the one hand, the progress in systemic therapy has facilitated the postponement of local treatment for BM. Conversely, it has also introduced a new viewpoint regarding radiation-induced necrosis (RN), which may be influenced by the timing of these systemic treatment regimens with RT.

Recent findings highlight an elevated risk of severe radiation necrosis (RN) associated with antibody-drug conjugates (ADCs) such as T-DM1 and T-DXd when given concurrently with cranial radiotherapy (CNS-RT), defined as administration within a 4-week window prior to or following radiation. Data from a recent trial indicated that symptomatic RN occurred in 27% of patients in the concurrent treatment cohort, compared to just 7% in the non-concurrent cohort (p = 0.014) (107). In contrast, the DESTINY-Breast03 trial did not categorize RN as an adverse event of special interest, largely due to the prohibition of radiotherapy during the study period and the absence of information on prescribed treatment following CNS progression (25, 26).

In the context of Tucatinib in combination with RT, the HER2CLIMB trial required a minimum interval for the inclusion of patients with prior CNS-RT and permitted treatment interruptions of up to 6 weeks to facilitate local therapy for CNS progression. The regimen utilized in HER2CLIMB (Tucatinib, Capecitabine, and trastuzumab) can be re-administered a minimum of 7 days post-SRS or 21 days after WBRT (28).

Looking ahead, further research is needed to conduct a comprehensive analysis of salvage CNS-RT and its association with RN incidence, aiming to optimize radiotherapy strategies for CNS progression in the context of ADC treatment.

In this evolving therapeutic scenario, early detection of BM has a fundamental role. Enhanced comprehension of the mechanisms underlying BM and thorough molecular characterisation of BM could offer valuable assistance in finding more selective and effective strategies for early detection and treatment of BM.

Such objectives could benefit from the new and more sophisticated tumour analysis methods and circulating biomarkers detection.

Circulating biomarkers such as tumour nucleic acids, CTCs, and EVs offer insights into tumour evolution, particularly for tracking treatment response and disease advancement. While further refinement in biomarker detection techniques is necessary, these tools have already proven their diagnostic, prognostic, and predictive significance across various tumour types, including breast cancer. Additionally, they could be utilised in preclinical settings to explore mechanisms of acquired drug resistance, tumour invasion, and spread.
